# (*E*)-*N*′-[(*E*)-3-Phenyl­allyl­idene]benzo­hydrazide

**DOI:** 10.1107/S1600536811053645

**Published:** 2011-12-17

**Authors:** Gui-Ming Deng, Zhen Chen, Chao-Run Wang, He-Ming Zhang

**Affiliations:** a MOE Key Laboratory of Laser Life Science & Institute of Laser Life Science, College of Biophotonics, South China Normal University, Guangzhou 510631, People’s Republic of China; b The First Affiliated Hospital of Hunan University of Chinese Medicine, Changsha, 410007, People’s Republic of China

## Abstract

In the title mol­ecule, C_16_H_14_N_2_O, the dihedral angle between the two phenyl rings is 23.5 (6)°. In the crystal, N—H—O hydrogen bonds link mol­ecules into chains running along the *a* axis.

## Related literature

For general background to the applications of Schiff bases in the pharmaceutical and agrochemical fields, see: Bernardino *et al.* (2006 ▶); Zhang *et al.* (2008 ▶). For related structures, see: Ji & Shi (2008 ▶); He & Liu (2005 ▶); Zhen & Han (2005 ▶); Zhang *et al.* (2007 ▶).
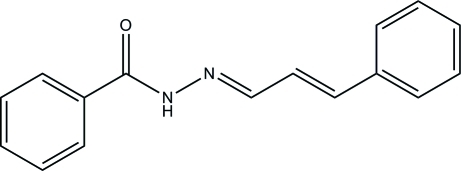


## Experimental

### 

#### Crystal data

C_16_H_14_N_2_O
*M* *_r_* = 250.29
Orthorhombic, 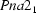

*a* = 8.427 (3) Å
*b* = 10.439 (4) Å
*c* = 15.724 (6) Å
*V* = 1383.2 (9) Å^3^
*Z* = 4
Mo *K*α radiation
μ = 0.08 mm^−1^
*T* = 296 K
0.16 × 0.13 × 0.10 mm

#### Data collection

Bruker APEXII CCD diffractometer
6868 measured reflections
1761 independent reflections
1361 reflections with *I* > 2σ(*I*)
*R*_int_ = 0.040

#### Refinement

*R*[*F* ^2^ > 2σ(*F* ^2^)] = 0.041
*wR*(*F* ^2^) = 0.096
*S* = 1.01
1761 reflections
176 parameters
1 restraint
H atoms treated by a mixture of independent and constrained refinement
Δρ_max_ = 0.09 e Å^−3^
Δρ_min_ = −0.12 e Å^−3^

### 

Data collection: *SMART* (Bruker, 2001 ▶); cell refinement: *SAINT-Plus* (Bruker, 2003 ▶); data reduction: *SAINT-Plus*; program(s) used to solve structure: *SHELXTL* (Sheldrick, 2008 ▶); program(s) used to refine structure: *SHELXTL*; molecular graphics: *SHELXTL* (Sheldrick, 2008 ▶); software used to prepare material for publication: *SHELXTL*.

## Supplementary Material

Crystal structure: contains datablock(s) I, global. DOI: 10.1107/S1600536811053645/cv5216sup1.cif

Structure factors: contains datablock(s) I. DOI: 10.1107/S1600536811053645/cv5216Isup2.hkl

Supplementary material file. DOI: 10.1107/S1600536811053645/cv5216Isup3.cml

Additional supplementary materials:  crystallographic information; 3D view; checkCIF report

## Figures and Tables

**Table 1:** Hydrogen-bond geometry (Å, °)

*D*—H⋯*A*	*D*—H	H⋯*A*	*D*⋯*A*	*D*—H⋯*A*
N1—H1*A*⋯O1^i^	0.88 (2)	2.05 (2)	2.898 (3)	161 (2)

Symmetry code: (i) 
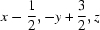
.

**Fig. 1.**
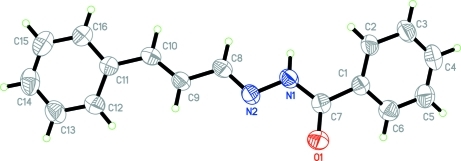
The molecular structure of (I) showing the atomic numbering and 30% probability displacement ellipsoids.

**Fig. 2.**
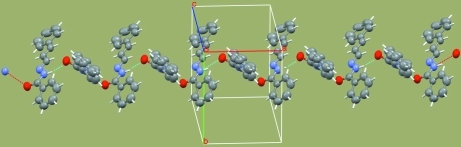
A portion of the packing, viewed down the a axis. The dashed lines represent the intermolecular hydrogen bonds.

## supplementary crystallographic information

### Comment

Schiff bases have attracted much attention due to their various activities in pharmaceutical and agrochemical fields (Bernardino *et al.*, 2006; Zhang *et al.*, 2008). We now report the synthesis and structure of the title compound, (I).

In (I) (Fig. 1), the Schiff base molecule adopts an *E* geometry with respect to the C=N bond. All bond lengths and angles are normal and comparable with those found in the related compounds (Ji *et al.*, 2008; He *et al.*., 2005; Zhen *et al.*, 2005; Zhang *et al.*., 2007). The dihedral angle between the two benzene rings is 23.5 (6)°.

In the crystal structure,  intermolecular N—H—O hydrogen bonds (Table 1) link  molecules into chains running along the *a* axis (Fig. 2).

### Experimental

The title compound was synthesized by the reaction of benzoylhydrazine (1 mmol, 136.2 mg) with 3-phenyl-propenal (1 mmol, 132.2 mg) in ethanol (20 ml) under reflux conditions (348 K) for 6 h. The solvent was removed and the solid product recrystallized from tetrahydrofuran. After two days yellow crystals suitable for X-ray diffraction study were obtained. Yield, 222.7 mg, 83%. Analysis calculated for C_16_H_14_N_2_O: C 76.78, H 5.64, N 11.19%; found: C 76.73, H 5.61, N 11.21%.

### Refinement

The H1A atom bonded to N1 was located in a difference map and refined isotropically, other H atoms were placed in geometrically idealized positions and allowed to ride on their parent atoms,*C*—H=0.93, with *U*_iso_(H)=1.2*U*_eq_(C). In the absence of any significant anomalous scatterers in the molecule, the 1215  Friedel pairs were merged before the final refinement.

### Crystal data

**Table:**

C_16_H_14_N_2_O	*F*(000) = 528.0
*M**_r_* = 250.29	*D*_x_ = 1.202 Mg m^−^^3^
Orthorhombic, *P**n**a*2_1_	Mo *K*α radiation, λ = 0.71073 Å
Hall symbol: P 2c -2n	Cell parameters from 968 reflections
*a* = 8.427 (3) Å	θ = 0.5–3.2°
*b* = 10.439 (4) Å	µ = 0.08 mm^−^^1^
*c* = 15.724 (6) Å	*T* = 296 K
*V* = 1383.2 (9) Å^3^	Block, yellow
*Z* = 4	0.16 × 0.13 × 0.10 mm

### Data collection

**Table:**

Bruker APEXII CCD diffractometer	1361 reflections with *I* > 2σ(*I*)
Radiation source: fine-focus sealed tube	*R*_int_ = 0.040
graphite	θ_max_ = 28.2°, θ_min_ = 2.3°
phi and ω scans	*h* = −11→11
6868 measured reflections	*k* = −12→13
1761 independent reflections	*l* = −14→20

### Refinement

**Table:**

Refinement on *F*^2^	Primary atom site location: structure-invariant direct methods
Least-squares matrix: full	Secondary atom site location: difference Fourier map
*R*[*F*^2^ > 2σ(*F*^2^)] = 0.041	Hydrogen site location: inferred from neighbouring sites
*wR*(*F*^2^) = 0.096	H atoms treated by a mixture of independent and constrained refinement
*S* = 1.01	*w* = 1/[σ^2^(*F*_o_^2^) + (0.0355*P*)^2^] where *P* = (*F*_o_^2^ + 2*F*_c_^2^)/3
1761 reflections	(Δ/σ)_max_ < 0.001
176 parameters	Δρ_max_ = 0.09 e Å^−^^3^
1 restraint	Δρ_min_ = −0.12 e Å^−^^3^

### Special details

**Table:**

Geometry. All e.s.d.'s (except the e.s.d. in the dihedral angle between two l.s. planes) are estimated using the full covariance matrix. The cell e.s.d.'s are taken into account individually in the estimation of e.s.d.'s in distances, angles and torsion angles; correlations between e.s.d.'s in cell parameters are only used when they are defined by crystal symmetry. An approximate (isotropic) treatment of cell e.s.d.'s is used for estimating e.s.d.'s involving l.s. planes.
Refinement. Refinement of *F*^2^ against ALL reflections. The weighted *R*-factor *wR* and goodness of fit *S* are based on *F*^2^, conventional *R*-factors *R* are based on *F*, with *F* set to zero for negative *F*^2^. The threshold expression of *F*^2^ > σ(*F*^2^) is used only for calculating *R*-factors(gt) *etc*. and is not relevant to the choice of reflections for refinement. *R*-factors based on *F*^2^ are statistically about twice as large as those based on *F*, and *R*- factors based on ALL data will be even larger.

### Fractional atomic coordinates and isotropic or equivalent isotropic displacement parameters (Å^2^)

**Table:**

	*x*	*y*	*z*	*U*_iso_*/*U*_eq_	
C1	1.0668 (3)	0.6483 (2)	0.48249 (16)	0.0652 (6)	
C2	0.9616 (3)	0.7307 (2)	0.44374 (17)	0.0759 (7)	
H2	0.9252	0.8027	0.4727	0.091*	
C3	0.9099 (3)	0.7064 (3)	0.36146 (18)	0.0946 (9)	
H3	0.8373	0.7612	0.3357	0.114*	
C4	0.9659 (4)	0.6012 (4)	0.3178 (2)	0.0980 (9)	
H4	0.9308	0.5851	0.2628	0.118*	
C5	1.0721 (4)	0.5212 (3)	0.3549 (2)	0.0919 (8)	
H5	1.1107	0.4508	0.3251	0.110*	
C6	1.1229 (3)	0.5441 (3)	0.4371 (2)	0.0832 (7)	
H6	1.1957	0.4889	0.4622	0.100*	
C7	1.1258 (3)	0.6639 (2)	0.57099 (17)	0.0686 (6)	
C8	0.9802 (3)	0.8064 (2)	0.75525 (16)	0.0697 (7)	
H8	0.8937	0.8469	0.7302	0.084*	
C9	1.0068 (3)	0.8183 (2)	0.84500 (16)	0.0689 (7)	
H9	1.0972	0.7808	0.8680	0.083*	
C10	0.9088 (3)	0.8803 (2)	0.89690 (17)	0.0720 (7)	
H10	0.8252	0.9235	0.8710	0.086*	
C11	0.9157 (3)	0.8892 (2)	0.98938 (17)	0.0670 (6)	
C12	1.0264 (3)	0.8252 (3)	1.03800 (18)	0.0859 (8)	
H12	1.1026	0.7750	1.0112	0.103*	
C13	1.0268 (4)	0.8341 (3)	1.1250 (2)	0.1066 (10)	
H13	1.1025	0.7899	1.1565	0.128*	
C14	0.9169 (5)	0.9073 (3)	1.1655 (2)	0.1060 (11)	
H14	0.9170	0.9130	1.2245	0.127*	
C15	0.8062 (4)	0.9724 (3)	1.1190 (2)	0.1051 (11)	
H15	0.7315	1.0233	1.1465	0.126*	
C16	0.8049 (3)	0.9630 (2)	1.03158 (18)	0.0849 (8)	
H16	0.7284	1.0070	1.0006	0.102*	
H1A	0.938 (3)	0.761 (2)	0.6107 (14)	0.062 (7)*	
N1	1.0322 (3)	0.7305 (2)	0.62472 (13)	0.0713 (6)	
N2	1.0757 (2)	0.7398 (2)	0.70954 (13)	0.0714 (6)	
O1	1.25170 (19)	0.61572 (17)	0.59406 (13)	0.0867 (5)	

### Atomic displacement parameters (Å^2^)

**Table:**

	*U*^11^	*U*^22^	*U*^33^	*U*^12^	*U*^13^	*U*^23^
C1	0.0605 (13)	0.0711 (14)	0.0640 (16)	−0.0100 (13)	0.0033 (13)	0.0047 (13)
C2	0.0778 (15)	0.0902 (17)	0.0597 (16)	0.0012 (13)	0.0077 (16)	0.0089 (15)
C3	0.094 (2)	0.125 (2)	0.064 (2)	0.0053 (19)	−0.0004 (17)	0.0212 (19)
C4	0.108 (2)	0.125 (2)	0.0611 (19)	−0.021 (2)	0.0000 (18)	0.0016 (19)
C5	0.107 (2)	0.0881 (18)	0.080 (2)	−0.0092 (18)	−0.0003 (17)	−0.0115 (18)
C6	0.0847 (16)	0.0818 (16)	0.083 (2)	−0.0035 (14)	−0.0061 (17)	−0.0025 (16)
C7	0.0635 (14)	0.0725 (15)	0.0697 (18)	−0.0098 (13)	−0.0013 (14)	0.0004 (14)
C8	0.0603 (14)	0.0779 (17)	0.0708 (18)	−0.0045 (13)	−0.0098 (14)	0.0068 (14)
C9	0.0631 (14)	0.0798 (17)	0.0639 (17)	−0.0003 (13)	−0.0082 (13)	0.0039 (13)
C10	0.0632 (15)	0.0773 (16)	0.0756 (18)	0.0023 (14)	−0.0107 (14)	0.0051 (14)
C11	0.0671 (14)	0.0620 (13)	0.0718 (18)	−0.0046 (13)	−0.0022 (15)	0.0013 (12)
C12	0.0867 (18)	0.0919 (18)	0.079 (2)	0.0115 (17)	−0.0065 (16)	0.0057 (18)
C13	0.139 (3)	0.106 (2)	0.075 (2)	0.012 (2)	−0.015 (2)	0.011 (2)
C14	0.166 (3)	0.0823 (19)	0.069 (2)	−0.018 (2)	0.009 (2)	0.0045 (17)
C15	0.138 (3)	0.081 (2)	0.097 (3)	0.000 (2)	0.030 (2)	−0.0086 (18)
C16	0.0878 (19)	0.0784 (16)	0.088 (2)	0.0029 (16)	0.0050 (16)	−0.0050 (15)
N1	0.0655 (13)	0.0850 (15)	0.0634 (15)	−0.0003 (12)	−0.0073 (13)	0.0041 (12)
N2	0.0702 (12)	0.0839 (13)	0.0601 (14)	−0.0021 (11)	−0.0045 (11)	0.0042 (10)
O1	0.0694 (10)	0.1060 (12)	0.0847 (13)	0.0098 (10)	−0.0118 (10)	−0.0070 (10)

### Geometric parameters (Å, °)

**Table:**

C1—C2	1.378 (3)	C9—C10	1.330 (3)
C1—C6	1.384 (3)	C9—H9	0.9300
C1—C7	1.486 (3)	C10—C11	1.458 (3)
C2—C3	1.389 (4)	C10—H10	0.9300
C2—H2	0.9300	C11—C12	1.379 (4)
C3—C4	1.378 (4)	C11—C16	1.380 (4)
C3—H3	0.9300	C12—C13	1.371 (4)
C4—C5	1.356 (4)	C12—H12	0.9300
C4—H4	0.9300	C13—C14	1.359 (5)
C5—C6	1.382 (4)	C13—H13	0.9300
C5—H5	0.9300	C14—C15	1.366 (4)
C6—H6	0.9300	C14—H14	0.9300
C7—O1	1.229 (3)	C15—C16	1.378 (4)
C7—N1	1.349 (3)	C15—H15	0.9300
C8—N2	1.284 (3)	C16—H16	0.9300
C8—C9	1.434 (3)	N1—N2	1.386 (3)
C8—H8	0.9300	N1—H1A	0.88 (2)
			
C2—C1—C6	118.9 (3)	C8—C9—H9	118.4
C2—C1—C7	124.1 (2)	C9—C10—C11	128.2 (2)
C6—C1—C7	117.0 (2)	C9—C10—H10	115.9
C1—C2—C3	120.0 (3)	C11—C10—H10	115.9
C1—C2—H2	120.0	C12—C11—C16	117.5 (3)
C3—C2—H2	120.0	C12—C11—C10	123.3 (3)
C4—C3—C2	120.2 (3)	C16—C11—C10	119.2 (3)
C4—C3—H3	119.9	C13—C12—C11	121.5 (3)
C2—C3—H3	119.9	C13—C12—H12	119.3
C5—C4—C3	120.2 (3)	C11—C12—H12	119.3
C5—C4—H4	119.9	C14—C13—C12	120.2 (3)
C3—C4—H4	119.9	C14—C13—H13	119.9
C4—C5—C6	120.0 (3)	C12—C13—H13	119.9
C4—C5—H5	120.0	C13—C14—C15	119.7 (3)
C6—C5—H5	120.0	C13—C14—H14	120.2
C5—C6—C1	120.8 (3)	C15—C14—H14	120.2
C5—C6—H6	119.6	C14—C15—C16	120.2 (3)
C1—C6—H6	119.6	C14—C15—H15	119.9
O1—C7—N1	122.1 (2)	C16—C15—H15	119.9
O1—C7—C1	121.3 (2)	C15—C16—C11	120.9 (3)
N1—C7—C1	116.6 (2)	C15—C16—H16	119.5
N2—C8—C9	120.0 (2)	C11—C16—H16	119.5
N2—C8—H8	120.0	C7—N1—N2	119.0 (2)
C9—C8—H8	120.0	C7—N1—H1A	123.6 (15)
C10—C9—C8	123.3 (2)	N2—N1—H1A	116.9 (15)
C10—C9—H9	118.4	C8—N2—N1	114.3 (2)
			
C6—C1—C2—C3	2.0 (3)	C9—C10—C11—C12	−3.7 (4)
C7—C1—C2—C3	−178.3 (2)	C9—C10—C11—C16	177.5 (2)
C1—C2—C3—C4	−1.2 (4)	C16—C11—C12—C13	0.2 (4)
C2—C3—C4—C5	−0.2 (4)	C10—C11—C12—C13	−178.7 (3)
C3—C4—C5—C6	0.8 (4)	C11—C12—C13—C14	−0.2 (5)
C4—C5—C6—C1	0.0 (4)	C12—C13—C14—C15	−0.2 (5)
C2—C1—C6—C5	−1.4 (3)	C13—C14—C15—C16	0.7 (5)
C7—C1—C6—C5	178.8 (2)	C14—C15—C16—C11	−0.7 (5)
C2—C1—C7—O1	−156.8 (2)	C12—C11—C16—C15	0.3 (4)
C6—C1—C7—O1	23.0 (3)	C10—C11—C16—C15	179.1 (2)
C2—C1—C7—N1	24.8 (3)	O1—C7—N1—N2	−3.2 (3)
C6—C1—C7—N1	−155.5 (2)	C1—C7—N1—N2	175.23 (19)
N2—C8—C9—C10	−176.6 (2)	C9—C8—N2—N1	176.9 (2)
C8—C9—C10—C11	174.1 (2)	C7—N1—N2—C8	179.2 (2)

### Hydrogen-bond geometry (Å, °)

**Table:**

*D*—H···*A*	*D*—H	H···*A*	*D*···*A*	*D*—H···*A*
N1—H1A···O1^i^	0.88 (2)	2.05 (2)	2.898 (3)	161 (2)

Symmetry codes: (i) *x*−1/2, −*y*+3/2, *z*.
